# Endoscopic Bariatric and Metabolic Therapies and Their Effects on Metabolic Syndrome and Non-alcoholic Fatty Liver Disease - A Systematic Review and Meta-Analysis

**DOI:** 10.3389/fmed.2022.880749

**Published:** 2022-05-09

**Authors:** Shi-Yan Lee, Haoxing Lai, Yang Jie Chua, Min Xian Wang, Guan-Huei Lee

**Affiliations:** ^1^Gastroenterology & Hepatology, National University Hospital, Singapore, Singapore; ^2^Department of Medicine, Yong Loo Lin School of Medicine, National University of Singapore, Singapore, Singapore; ^3^Saw Swee Hock School of Public Health, National University of Singapore and National University Health System, Singapore, Singapore; ^4^Centre of Infectious Disease Epidemiology and Research, National University of Singapore, Singapore, Singapore

**Keywords:** non-alcoholic steatohepatitis, diabetes mellitus, hypertension, dyslipidemia, atherosclerosis

## Abstract

**Background:**

Endoscopic bariatric and metabolic therapies (EBMTs) are procedures that utilize instruments that require flexible endoscopy or placement of devices for inducing weight loss. We perform a systematic review and meta-analysis to evaluate four modalities – intragastric balloon (IGB), endoscopic sleeve gastroplasty (ESG), duodeno-jejunal bypass liner (DJBL), and duodenal mucosa resurfacing (DMR), for their efficacy and safety on weight loss, non-alcoholic fatty liver disease, and metabolic syndrome.

**Methods:**

Databases MEDLINE *via* PubMed, and EMBASE are searched and relevant publications up to January 26, 2022 are assessed. Studies are included if they involved human participants diagnosed with obesity and obesity-related comorbid conditions who are treated with any of the 4 EBMTs. IGB and DJBL were chosen as the interventions for the meta-analysis with weight loss (percentage total body weight loss or body mass index) and glycemic control (fasting plasma glucose or HbA1c) as the two main outcomes analyzed.

**Results:**

Six hundred and forty-eight records are reviewed, of which 15 studies are found to be duplicates. Of the 633 records screened, 442 studies are excluded. One hundred and ninety-one articles are assessed for eligibility, for which 171 are excluded. A total of 21 publications are included. Twelve studies are on IGB, two studies on ESG, five studies on DJBL, and two studies on DMR. In these studies with appropriate control, IGB, ESG, and DJBL showed promising benefits on weight loss reduction compared to standard medical therapy (SMT), while DMR appeared to have the least weight reduction benefit. However, the impact on glycemic control featured more prominently in DMR as compared to the rest of the modalities. Different EBMTs have different adverse effect profiles, although device-related adverse events are featured more prominently in DJBL. In the IGB group, there was a significant reduction in 6-month %TBWL [weighted mean difference (WMD) 5.45 (3.88, 7.05)] and FPG WMD −4.89 mg/dL (−7.74, −2.04) compared to the SMT group. There was no significant reduction in BMI between the DJBL and SMT group WMD −2.73 (−5.52, 0.07) kg/m^2^.

**Conclusion:**

EBMTs have demonstrated a significant impact on weight loss and metabolic comorbidities, and reasonable safety profiles in the studies reviewed. Some data is available to demonstrate reduction of hepatic steatosis, but there is no high-quality data supporting benefits on hepatic lobular inflammation or fibrosis.

## Introduction

Proliferating rapidly over the past few decades, metabolic syndrome has become a major global epidemic and is a significant contributor to morbidity and mortality around the globe (1). Metabolic syndrome was formally defined by the World Health Organization (WHO) in 1999 as glucose intolerance, impaired glucose tolerance (IGT) or diabetes mellitus (DM), and/or insulin resistance, together with two or more of the components: raised arterial pressure, plasma triglyceride and or low HDL-C, central obesity, microalbuminuria (2).

Although the actual definition of this entity is heavily debated, the consequence of metabolic syndrome is undisputed. Metabolic syndrome leads to increased risk for obesity-related comorbidities such as cardiovascular disease (3), chronic renal disease (4), certain types of cancers (such as breast, colorectal, endometrial cancer) (5), and non-alcoholic fatty liver disease (NAFLD), which are leading causes of preventable death among adults. Among them, NAFLD is the most common cause of chronic liver disease worldwide, affecting ~25% of the global population (6). NAFLD is exacerbated by often interdependent metabolic syndrome parameters such as central obesity, insulin resistance, and elevated plasma triglyceride. These obesity-related comorbid parameters are thought to increase the accumulation of fat in the liver *via* the suppression of lipolysis and stimulation of *de novo* lipogenesis, which then promotes lipotoxicity and the activation of Kupffer cells, progressively leading to hepatic fibrogenesis, simple steatosis, steatohepatitis and eventually advanced cirrhosis (7).

Patients with metabolic syndrome are often prescribed standard medical therapy (SMT), which includes lifestyle modifications such as dietary alterations, physical exercise and pharmacological management. However, compliance and efficacy are often poor. Weight loss by the means of SMT alone is likely inadequate to effectively treat the rising incidences of these obesity-related comorbidities, particularly since less than 10 percent of patients are able to achieve at least 10 percent of weight loss (8) to ensure major improvement in the histological features of fatty-liver fibrosis (8, 9).

While bariatric surgery is well known to be one of the most effective treatment modalities for morbidly obese patients, it is invasive and associated with short term (bleeding, infection, and other perioperative risks), and long-term surgical risks. As a result, only one percent of the eligible patients choose to undergo bariatric surgery for obesity in the United States annually (10). This has prompted the development of newer, yet minimally invasive treatment armamentarium for weight loss – endoscopic bariatric and metabolic therapies (EBMTs).

EBMTs are devices that require flexible endoscopy for placement or removals and are used as an alternative treatment for patients who do not qualify for or do not wish to undergo bariatric surgery. EBMTs are prized for their reversibility (12), short procedure time, technical ease, and lower adverse event rates and complications such as liver abscess, anastomotic leakage and bleeding (13).

In this review, we focus on four main EBMTs - intragastric balloon (IGB), duodenal-jejunal bypass liner (DJBL), duodenal mucosal resurfacing (DMR) and endoscopic sleeve gastroplasty (ESG) (11).

IGB was one of the earliest endoscopic bariatric therapies developed. It works by the temporary endoscopy-assisted introduction of a balloon into the stomach, which can be inflated with either air or liquid solution to different volumes to achieve the feeling of satiety, which eventually leads to reduced caloric consumption. DMR and DJBL are performed with endoscopic and fluoroscopic guidance. DMR targets the post papillary duodenal mucosa using hydrothermal ablation which results in alterations in the mucosa and absorptive properties. Submucosal expansion is first performed, followed by progressive ablation of the duodenal mucosa distal to the Ampulla of vater. On the other hand, DJBL involves the endoscopic insertion of an impermeable, fluoropolymer sleeve into the duodenum and proximal jejunum for up to 12 months. This prevents further digestion and absorption of gastric contents as it passes directly from the pylorus into the mid jejunum. Finally, ESG uses a miniature suturing device introduced endoscopically to create folds in the mucosa which reduces total gastric volume and aims to achieve similar results as sleeve gastrectomy without undergoing laparoscopic surgery.

This systematic review and meta-analysis aims to summarize and analyze existing data available on these four main EBMT modalities (IGB, ESG, DJBL and DMR) and their impact on metabolic syndrome parameters (weight change and BMI) and all-cause obesity-related metabolic comorbidities such as glycemic control and NAFLD. Our main focus was on high quality studies, predominantly randomized controlled trials with appropriate control arms i.e., standard medical therapy (SMT).

## Materials and Methods

### Eligibility Criteria

We included clinical studies published between 2012 and 26 January, 2022 which assessed the impact of EBMTs on metabolic syndrome and obesity-related comorbid conditions in adult human participants (Table 1 for PICOS criteria). We only included studies that had control with SMT, which comprised cohort studies and randomized controlled trials. We excluded studies without a control arm and those that had inappropriate control groups e.g., laparoscopic bariatric surgery. Abstracts and studies in non-English literature were excluded. Duplicates, or studies lacking original data were also excluded. This systematic review was further developed in accordance with the Preferred Items for Systematic Review and Meta-Analysis Reports (PRISMA) guideline.

**Table 1:** Participants, interventions, comparisons, outcomes and study design criteria used to define the research question for this systematic review.

**Variable**	**Description**
Population	Humans diagnosed with obesity and obesity-related comorbid conditions (e.g., CVD, NAFLD, T2DM)
Intervention	Endoscopic Bariatric and Metabolic Therapies, with our review restricted to 4 key modalities of IGB, ESG, DJBL and DMR; no restrictions on the duration, demographics and regime of each modality selected
Comparator	Randomized controlled trials/Cohort studies: Standard medical treatment (e.g., lifestyle therapy and pharmacotherapy)
Outcome	Obesity parameters (weight change and BMI) and all-cause obesity-related comorbid condition parameters (e.g., glycemic control, cardiometabolic risk factors and liver biochemistry)
Study design	Randomized controlled trials, Comparative cohort studies
Research question	Do Endoscopic Bariatric and Metabolic Therapies have an effect on Metabolic Syndrome parameters and all-cause obesity-related comorbid condition parameters, compared to standard medical treatment?

*CVD, cardiovascular disease; NAFLD, non-alcoholic fatty liver disease; T2DM, type 2 diabetes mellitus; IGB, intragastric balloon; ESG, endoscopic sleeve gastroplasty; DJBL, duodenal-jejunal bypass liner; DMR, duodenal mucosal resurfacing; BMI, body mass index*.

### Information Sources

From the conception of this systematic review to 26 January, 2022, a comprehensive search of two electronic databases was conducted to identify all relevant articles. These databases were MEDLINE *via* PubMed, and EMBASE. Our search terms included both text and medical subject headings where appropriate. Modifications of literature search strategies to suit each database were also performed.

For instance, on PubMed, a combination of the following medical subject heading terms “Cardiovascular diseases OR “atherosclerosis” OR “metabolic syndrome” AND “gastric balloon” were used to search for studies evaluating the impact of IGBs on obesity-related comorbidities (Appendix). Our search was further limited by “Classical article,” “Clinical study,” “Clinical trial,” “Controlled clinical trial,” “Observational study,” “Randomized controlled trial,” “Review,” “Humans,” “Adults,” “English,” “Core clinical journals” and “MEDLINE.” The methods for data collection and analysis strategies were also based on the Cochrane Handbook of Systematic Reviews for Interventions.

### Selection of Studies, Data Collection and Summary Measures

**Figure 1:**
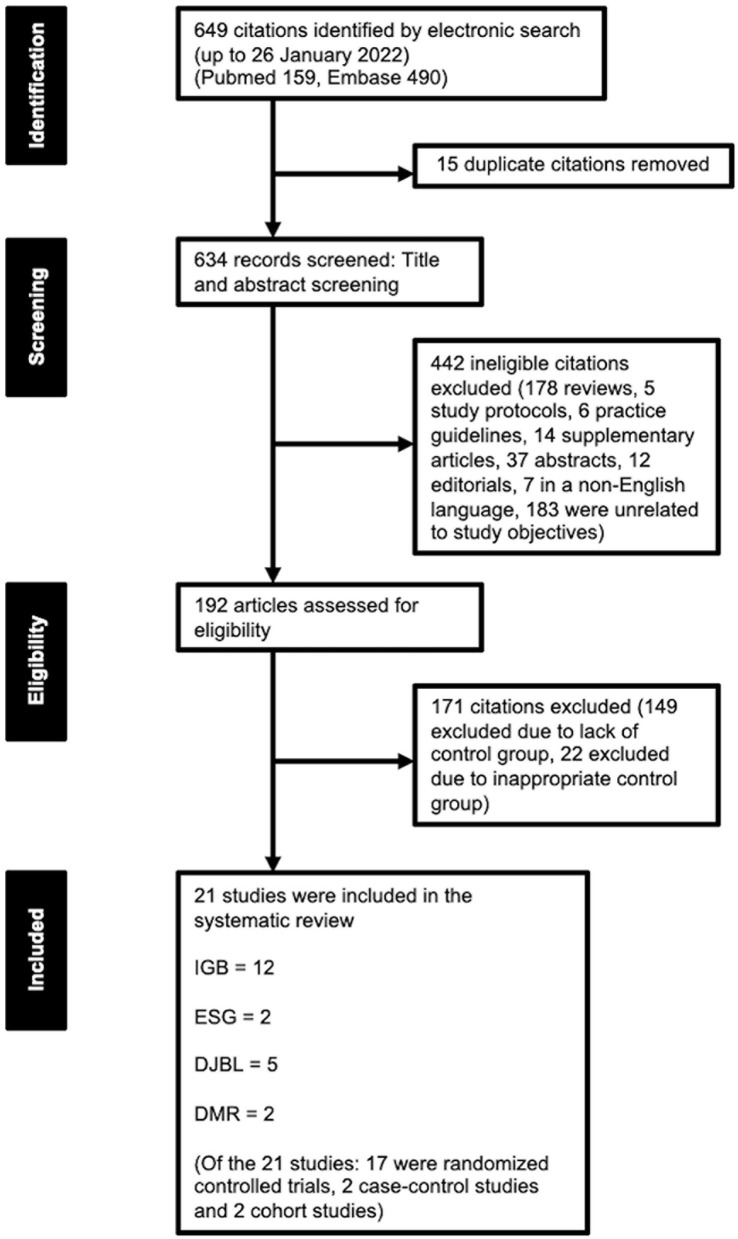
Summary of study selection process.

Two authors (CYJ and LHX) independently reviewed the relevant studies which were identified by the search process as described above. As summarized by Figure 1, full-text articles of all citations determined to meet the inclusion criteria were retrieved and duplicates were excluded. Each article was independently inspected to ensure inclusion criteria was met. The studies that were finally selected for the systematic review are included in Tables 2–**5**. Relevant study data were independently reviewed, selected and extracted. The primary outcome of interest is the effect on weight loss, while the secondary outcomes of interest are the effects on glycemic control, cardiometabolic risk factors (including blood pressure, lipid levels and cholesterol levels) and liver biochemistry.

**Table 2:** Summary of studies included in the meta-analysis.

**References**	**Type of study**	**No. of participants**	**Age (y)**	**Female (%)**	**Starting BMI (kg/m^**2**^)**	**Morbidity**	**Modality**	**Follow-up (mo)**	**Outcomes of interest**
Courcoulas et al. (21)	RCT (vs. SMT)	255	38.7 ± 9.37 (IGB), 40.8 ± 9.61 (SMT)	89.8	35.0	Obesity	IGB	6, 12	%TBWL
Sullivan et al. (22)	RCT (vs. SMT)	387	42.7 ± 9.6 (IGB), 42.5 ± 9.3(SMT)	88.1	35.2 ± 2.7 (IGB), 35.5 ± 2.7 (SMT)	Obesity	IGB	6	%TBWL, FPG
Ponce et al. (23)	RCT (vs. SMT)	326	43.8 ± 9.5 (IGB), 44.0 ± 10.2 (SMT)	95.1	35.3 ± 2.8 (IGB), 35.4 ± 2.6 (SMT)	Obesity	IGB	6	%TBWL
Fuller et al. (25)	RCT (vs. SMT)	66	43.4 ± 9.4 (IGB), 48.1 ± 7.3 (SMT)	66.7	36.0 ± 2.7 (IGB), 36.7 ± 2.9 (SMT)	Obesity, Metabolic syndrome	IGB	6, 12	%TBWL
Mariani et al. (28)	Prospective cohort study (vs. SMT)	32	40.81 ± 9.73 (IGB), 43.80 ± 10.36 (SMT)	59.3	41.82 ± 6.28 (IGB), 38.95 ± 6.90 (SMT)	Obesity	IGB	6	FPG
Ruban et al. (37)	RCT (vs. SMT)	170	51.6 ± 7.94 (DJBL), 51.9 ± 8.46 (SMT)	45.9	36.82 ± 4.955 (DJBL), 35.82 ± 4.222 (SMT)	Obesity, T2DM	DJBL	12	%HbA1c
Glaysher et al. (38)	RCT (vs. SMT)	170	51.6 ± 7.8 (DJBL), 52.3 ± 8.3 (SMT)	45.0	37.0 ± 5.0 (DJBL), 35.4 ± 3.7 (SMT)	Obesity, T2DM	DJBL	12	BMI
Caiazzo et al. (39)	RCT (vs. SMT)	80	48.1 (42.7–50.8) (DJBL), 46.7 (45.3–50.9) (SMT)	67.9	38.4 (36.8–39.9) (DJBL), 37.9 (36.3–39.5) (SMT)	Obesity, Metabolic syndrome	DJBL	12	%HbA1c
Laubner et al. (40)	Retrospective case-matched study (vs. SMT)	333	51.9 ± 9.0 (DJBL), 52.5 ± 16.2 (SMT)	58.9	42.6 ± 6.8 (DJBL), 41.9 ± 8.6 (SMT)	Obesity, T2DM	DJBL	12	BMI, %HbA1c
Koehestanie et al. (41)	RCT (vs. SMT)	77	49.5 [42-58] (DJBL), 49.0 [44-55] (SMT)	63.0	34.6 [32.4–38.1] (DJBL), 36.8 [32.6–42.0] (SMT)	Obesity, T2DM	DJBL	12	BMI

*Data presented as mean ± standard deviation or mean (interquartile range) or median [interquartile range]*.

*RCT, randomized controlled trial; IGB, intragastric balloon; DJBL, duodenal-jejunal bypass liner; SMT, standard medical therapy; T2DM, type 2 diabetes mellitus; %TBWL, percentage total body weight loss; FPG, fasting plasma glucose; %HbA1c, percentage glycated hemoglobin; BMI, body mass index*.

### Quality and Risk of Bias Assessment

Two independent reviewers utilized the Cochrane RoB 2 tool (14) to evaluate the risk of bias for RCTs and the Cochrane ROBINS-I tool (15) for non-randomized studies. RoB 2 includes five domains which are potential causes of bias: randomization, deviation from intended interventions, missing outcome data, outcome measurement and selection of reported results. Responses to the signaling questions within each domain are used by an algorithm to come to a judgment on the risk of bias for that domain, and the overall risk of bias for an outcome is determined by the judgment for individual domains. Each outcome can be assigned either a “low risk of bias,” “some concerns” or “high risk of bias” which indicates the strength of the evidence. ROBINS-1 is similar but includes four types of bias: confounding, selection, information and reporting. Each domain can be assessed to have a “low,” “moderate,” “serious” or “critical” risk of bias. Similarly, the assessments for the individual domains are used to come up with an overall risk of bias for the outcome, with a “low” risk of bias indicating that the study is comparable to a sound randomized trial. Any disagreements on the quality and risk of bias assessment were discussed and resolved by consensus (**Table 7**).

### Statistical Analysis

This study adopts an intention-to-treat (ITT) analysis as it is more representative of the real-world outcomes of the intervention (16). Thus, we assessed outcomes of the ITT analysis of the selected articles (Table 2). For studies with IGB interventions, we pooled the following outcomes: percentage of total body weight loss (%TBWL) at 6 months and 12 months from insertion, and fasting plasma glucose (FPG) at 6 months from insertion. For studies involving the DJBL, the following outcomes were pooled: change in body mass index (BMI), and percentage change in HbA1c levels (%HbA1c) at 12 months after insertion. IGB and DJBL were the chosen interventions for the meta-analysis given their potential to be implemented in the clinical setting, while these outcomes were the most commonly reported outcomes for these interventions.

For each group, the pre- and post-intervention means, standard deviations (SDs), change values, and SDs of the change values were extracted from studies when available. If unavailable, values were calculated from information provided in the study. The following calculations were undertaken in this study:

1. Change-value SDs for each group were calculated using 95% confidence intervals (CI), or *p*-values for the difference between pre-intervention and post-intervention means if the former was unavailable. The change value SDs for an outcome were calculated separately for IGB/DJBL and SMT groups for each study not reporting it (17).

2. The change in HbA1C levels for one study (38) was also converted from mmol/mol to percentage using the published formula by Jones et al. (18).

3. Finally, the mean difference in change value and its corresponding standard error (SE) between IGB/DJBL and SMT groups were calculated.

4. In 2 studies (25), the 95% CI of the mean difference of change between groups were used to calculate the SE of mean differences as change value SDs were not available and cannot be calculated from the published data (19).

For each outcome, the weighted mean differences (WMD) and 95% CIs was pooled with the metan command and random-effects model to account for between study variances (20). The I2 statistic and Cochran Q test was used to evaluate statistical heterogeneity, where heterogeneity was characterized as minimal (<25%), low (25–50%), moderate (50–75%) or high (>75%) and was significant if *p* < 0.05. All statistical tests were 2-sided with a statistical significance of *p* < 0.05, and performed using STATA 14.2.

## Results

### Search Results

A total of 649 records were reviewed, of which 15 duplicate studies were removed. Of the 649 records screened, 442 studies were excluded. 192 articles were assessed for eligibility, for which 171 were excluded (Figure 1).

After full-text review, a total of 21 publications satisfied all eligibility criteria and were included in this systematic review, which comprised 18 randomized controlled trials, 2 case-control studies and 2 cohort studies (Figure 1). Twelve studies were included for IGB only, two studies for ESG only, five studies for DJBL only, and two studies for DMR only.

All studies selected patients with obesity, of which five studies selected patients with obesity and type II DM, 1 study selected patients with obesity and non-alcoholic steatohepatitis, 1 study selected patients with obesity and a clinical diagnosis of metabolic syndrome, and 1 study selected female patients with obesity and polycystic ovary syndrome.

### Intragastric Balloon

#### Effect on Body Weight

**Figure 2:**
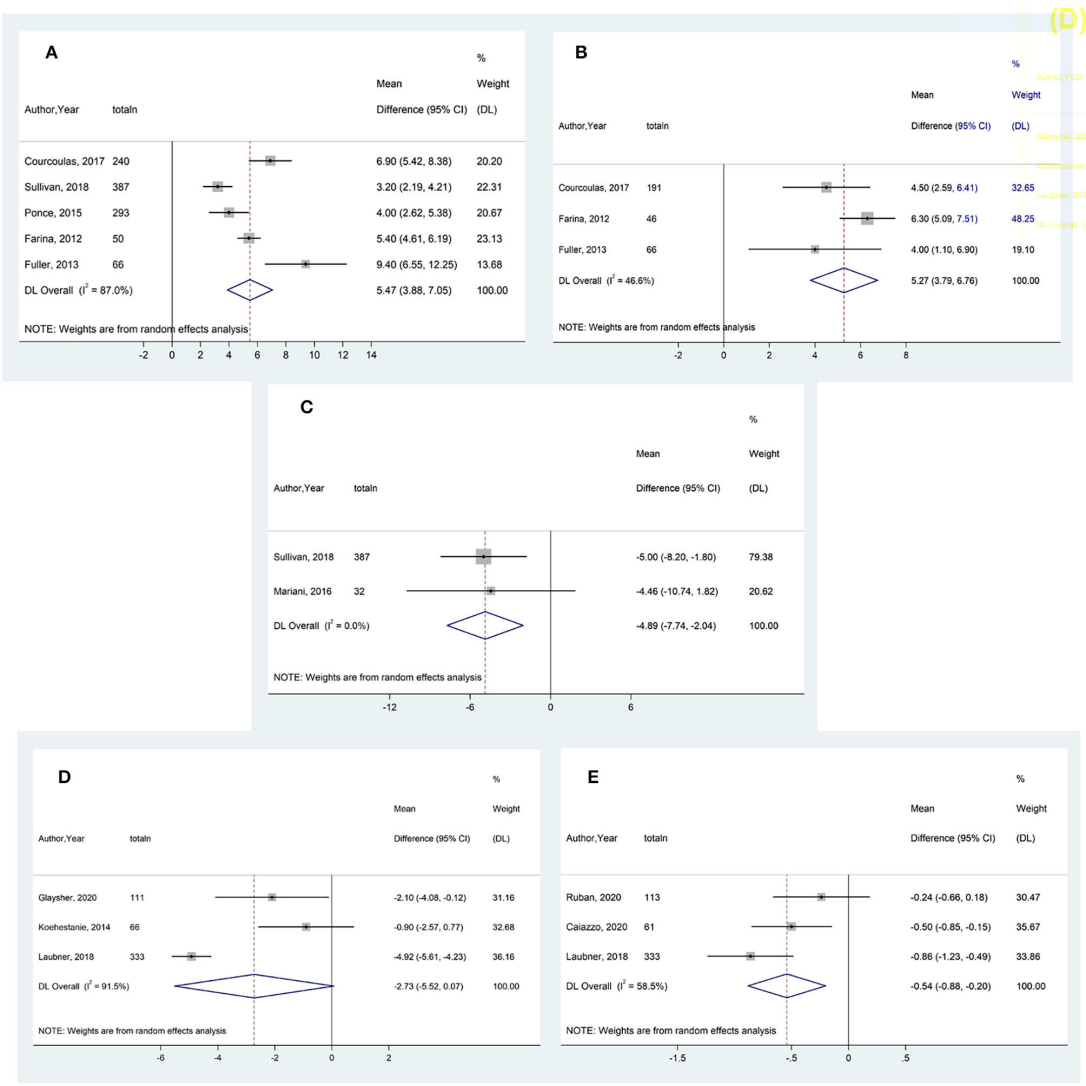
Weighted mean difference of **(A)** percentage total body weight loss (%TBWL) between IGB and SMT groups at 6 months, **(B)** %TBWL between IGB and SMT groups at 12 months, **(C)** reduction in fasting plasma glucose (mg/dL) between IGB and SMT groups at 6 months, **(D)** reduction in body mass index (kg/m^2^) between DJBL and SMT groups at 12 months, **(E)** reduction in percentage of glycated hemoglobin (%HbA1c) between DJBL and SMT groups at 12 months.

All twelve studies involving the IGB examined its efficacy in promoting weight loss as compared to SMT (Table 3). Ten were randomized controlled trials with the remaining being a cohort study. All but one of them found the IGB to be significantly more effective than SMT in promoting weight loss (21–32). In particular, 2 large randomized controlled trials by Courcoulas et al. (21) and Sullivan et al. (22), each with more than 200 participants, found the percentage of total body weight loss (%TBWL) to be significantly higher in the IGB group. In the Courcoulas study (21), weight loss was −3.3% of total body weight (−3.2 kg) in the SMT arm vs. −10.2% (−9.9 kg) in the IGB arm at the 6^th^ month. A third study by Ponce et al. (23) involving 326 participants also corroborates this benefit of weight loss, with the IGB group achieving 25.1% of excess weight loss (%EWL) vs. 11.3% in the SMT group (*p* = 0.004). Another study showed a significant decrease in median BMI in the IGB group (−2.8 kg/m^2^ vs. −0.4 kg/m2, *p* < 0.0001) at 6 months (24). Our meta-analysis, which included a total of five studies with 1,036 participants, found that there was a significant reduction in 6-month %TBWL in the IGB group, with a weighted mean difference (WMD) of 5.45 (3.88, 7.05) as compared to the SMT group (Figure 2A).

**Table 3:** Studies included for the study of IGB on Metabolic Syndrome and related comorbidities in adults.

**References**	**Type of study/No. of patients recruited**	**Study group(s)**	**Selection criteria/ Demographics of patients recruited**	**Results/outcome(s) of interest**
Courcoulas et al. (21)	Open label multicenter randomized controlled trial; *n* = 255 (IGB 125, SMT 130)	IGB vs. SMT	Patients with BMI ≥30 and ≤40 kg/m^2^, and a history of obesity for at least 2 years	%TBWL (6, 9, 12 mth): IGB >SMT (*p*? 0.001) Mean %EWL (9 mth): IGB 26.5% vs. SMT 9.7% (*p* = 0.32) FPG, lipids, BP (9 mth): no significant improvement
Sullivan et al. (22)	Double-blind randomized sham-controlled trial; *n* = 387 (IGB 198, SMT 189)	IGB vs. SMT	Patients with BMI ≥30 and ≤40 kg/m^2^	%TBWL (24 weeks): significantly greater in the IGB vs. SMT groups (6.6 vs. 3.4, *p* = 0.0354) Responder rate: significantly greater for the IGB group than the control group (*p* < 0.0001). FPG, lipids, SBP: decreased more significantly in the IGB group
Ponce et al. (23)	Randomized controlled trial; *n* = 326 (IGB 187, SMT 139)	IGB vs. SMT	Patients with BMI ≥30 and ≤40 kg/m^2^	%EWL (6 mth): IGB >SMT (25.1 vs. 11.3; *p* = 0.004) HbA1c, TG, LDL, SBP, DBP (24 weeks): IGB had significant improvement which persisted till 48 weeks (except for TG)
Coffin et al. (24)	Multicenter randomized controlled trial; *n* = 115 (IGB 55, SMT 60)	IGB vs. SMT	Patients with BMI >45 kg/m^2^	BMI decrease (6 mth): significantly greater in IGB group (2.8 vs. 0.4; *p* < 0.0001)
Fuller et al. (25)	Randomized controlled trial; *n* = 66 (IGB 37, SMT 37)	IGB vs. SMT	Patients with BMI ≥30 and ≤40 kg/m^2^	%TBWL (6 and 12 mth): IGB >SMT (14.2 vs. 4.8; *p* < 0.0001) and (9.2 vs. 5.2; *p* = 0.007) Metabolic syndrome remission (6 and 12 mth): greater in IGB than SMT (51.6% vs. 34.3%, n.s.) and (45.2% vs. 28.6%, n.s.)
Chan et al. (26)	Double-blind randomized controlled trial; *n* = 49 (IGB 26, SMT 23)	IGB vs. SMT	Patients with BMI ≥27 and ≤35 kg/m^2^	BMI (10 year): similar in IGB and control groups (p = 1.00). Weight maintenance; better in the IGB group (*p* = 0.05) FPG, lipids and SBP: not significantly different between groups
Mohammed et al. (27)	Randomized controlled trial; *n* = 128 (IGB 84, SMT 44)	IGB vs. SMT	Patients with BMI ≥30 kg/m^2^	%EWL (6 and 9 mth): IGB >SMT (21.5 vs. 4.20) and (27.3 and 5.30; *p*? 0.001) FPG and HOMA-IR (6 and 9 mth): greater decrease seen in IGB vs. SMT groups (*p*? 0.05)
Mariani et al. (28)	Prospective Cohort Study *n* = 32 (IGB 22, SMT 10)	IGB vs. SMT	Patients with BMI >30 kg/m^2^	%EBWL (6 mth): more significant in IGB group (33.73 vs. 22.08) HbA1c and DBP: significantly improved in the IGB but not the SMT group FPG, lipids and SBP: no significant decrease in both groups
Lee et al. (29)	Randomized controlled trial; *n* = 18 (IGB 8, SMT 10)	IGB vs. SMT	Patients with BMI ≥27 kg/m^2^ and who had histologic evidence of NASH	BMI decrease (6 mth): significantly greater in the IGB than control group (1.52 vs. 0.8; *p* = 0.0008) NAFLD activity scores (6 mth): significantly lower in the IGB group (2.0 vs. 4.0; *p* = 0.030). A trend toward improvement in the median steatosis scores was observed. No change in the median lobular inflammation, hepatocellular ballooning and fibrosis scores in both groups.
Vicente Martin et al. (30)	Randomized controlled trial; *n* = 66 (IGB 32, SMT 34)	IGB vs. SMT	Patients with BMI ≥40 kg/m^2^	%EWL and BMI reduction (6 mth): significantly greater in the IGB vs. SMT group (*p* < 0.001) 25% lost < 10% of their initial weight, and 9.4% lost < 5%
Farina et al. (31)	Randomized controlled trial; *n* = 50 (IGB 30, SMT 20)	IGB vs. SMT	Patients with BMI ≥30 and ?55 kg/m^2^	%TBWL (6 mth): IGB >SMT (14.5 vs. 9.1; *p* < 0.05) %EWL (6 mth): IGB >SMT (37.7 vs. 25.3; *p* < 0.05) Insulin sensitivity and triglyceride levels (12 mth): decrease in IGB group >for SMT (*p* < 0.05)
Takihata et al. (32)	Prospective cohort study; n=16 (IGB 8, SMT 8)	IGB vs. SMT	Patients with BMI >35 kg/m^2^	%EBWL and BMI reduction (6 mth): IGB less than SMT (p=0.248) HbA1c (6mth): Smaller improvement in IGB vs. SMT group (*p* = 0.073) FPG and lipids (6 mth): Not significantly different between groups

*%TBWL, percentage total body weight loss; %EWL, percentage excess weight loss; BMI, body mass index; FPG, fasting plasma glucose; HbA1C, glycated hemoglobin; BP, blood pressure; SBP, systolic blood pressure; DBP, diastolic blood pressure; TG, triglycerides; LDL, low density lipoprotein; HOMA-IR, homeostatic model assessment for insulin resistance; NAFLD, non-alcoholic fatty liver disease; NASH, non-alcoholic steatohepatitis*.

However, the durability of weight loss produced by the IGB may be limited, as both the Courcoulas and Sullivan studies saw an increase in participants' weight 6 months post-removal of the IGB. Fuller et al. (25) also reports this phenomenon, with %TBWL decreasing from 14.2 to 9.2% 3 months after removal of the IGB. Despite this, weight loss still remained more significant in the IGB group. Our meta-analysis corroborates this, with three studies involving a total of 303 participants reporting a slightly lower WMD in 12-month %TBWL of 5.27 (3.79, 6.76) between IGB and SMT groups (Figure 2B). A follow-up study of a randomized controlled trial by Chan et al. (26) also found that the BMI of the IGB and the control groups were similar at 10 years.

#### Effects on Glycemic Control and Cardiometabolic Risk Factors

*Six* studies examined the effect of IGB on HbA1c or fasting plasma glucose (FPG) compared to SMT (Table 2). Of these six studies, two studies (22, 28) which included a total of 419 participants analyzed the effect of IGB on FPG. Our analysis found that the IGB group experienced a slightly greater decrease in FPG, with the WMD in FPG at −4.89 mg/dL (−7.74, −2.04) (Figure 2C). The Sullivan study (22) found that FPG was significantly decreased in the IGB group as compared to the SMT group at 6 months, while there was no significant improvement in FPG in the Courcoulas study (21). Another smaller study by Mohammed et al. (27) found a significant reduction in FPG in the IGB arm compared to the SMT arm at 6 months which persisted till 9 months, while a cohort study by Mariani et al. (28) saw a significant decrease in HbA1c but not FPG at 6 months. In addition, the study by Chan et al. (26) found no significant difference in FPG at 10 years after IGB removal. Overall, the effect of IGB on glycemic control remains unclear.

Similarly, for other cardiometabolic risk factors such as hyperlipidemia and hypertension, Sullivan et al. (22) found a significant improvement in total cholesterol, plasma triglycerides and systolic blood pressure at 6 months over SMT. Fuller et al. (25) found a greater regression of metabolic syndrome parameters in the IGB arm, although it did not reach significance. These risk factors were again not significantly improved at removal and 3 months post-removal in the Courcoulas et al. (21) and Mariani et al. (28) studies respectively. The Chan et al. study (26) found no significant difference in FPG, total cholesterol, triglycerides and SBP at 10 years post-removal of the IGB.

#### Effects on NAFLD

A pilot study by Lee et al. randomized a group of obese patients with histologic evidence of NASH (29) into the BioEnterics Intragastric Balloon (*n* = 8) and the sham control groups, followed by a repeat liver biopsy after 24 weeks. A significant reduction in the mean BMI was observed in the IGB group (1.52 vs. 0.8; *p* = 0.0008) at the end of 24 weeks. Of note, the NAFLD activity scores were significantly lowered in the IGB group (2.0 vs. 4.0; *p* = 0.030). There was no change in the median lobular inflammation, hepatocellular ballooning, or fibrosis scores in both groups, though there was a trend toward improvement in the median steatosis score in the IGB group compared with the sham control group.

#### Safety

Adverse events in the IGB group are common but mild. The most common adverse symptoms include nausea, vomiting, and abdominal pain. About 20% of the subjects had their devices removed before 6 months because of an adverse event or subject request.

### Endoscopic Sleeve Gastroplasty

#### Effect on Body Weight

From our systematic review, we included two randomized controlled trials by Sullivan et al. (33) and Cheskin et al. (34) (Table 4). The Sullivan study found statistically significant weight loss that was almost 3.6 times more in the ESG group compared to the sham control group (4.95 vs. 1.38%) at 12 months. Responder rate (defined as subjects with at least 5% TBWL at 12 months) was 41.55% and 22.11% in active and sham groups respectively (*P* < 0.0001). The mean responder result was 11.5% of total body weight loss. Similar effects of ESG on body weight were corroborated by the Cheskin study (34).

**Table 4:** Studies included for the study of ESG on Metabolic Syndrome and related comorbidities in adults.

**References**	**Type of study/No. of patients recruited**	**Study group(s)**	**Selection criteria/ Demographics of patients recruited**	**Results/outcome(s) of interest**
Sullivan et al. (33)	Double-blind randomized sham-controlled trial; *n* = 332 (ESG 221, SMT 111)	ESG vs. SMT	Patients with BMI ≥30 and <35 kg/m^2^, with a history of obesity for at least 2 years assessed with at least one non-severe comorbid obesity-related condition; or BMI ≤35 kg/m^2^ and <40 kg/m^2^ with or without non-severe obesity-related comorbid condition	%TBWL (12 mth): significantly greater in the ESG vs. SMT groups (4.95 vs. 1.38, *p* < 0.0001) Responder rate of 5% TBWL: ESG significantly >control (41.55 vs. 22.11, *p* < 0.0001). FBG and improvement of T2DM (12 mth): ESG had significantly better improvement or resolution Hypertension, LDL, TG, and total cholesterol (12 mth): ESG had trends of greater reduction
Cheskin et al. (34)	Retrospective case-matched study; *n* = 386 (ESG 105, SMT 281)	ESG vs. SMT	Patients who underwent ESG with low-intensity diet and lifestyle therapy, and patients who underwent high-intensity diet and lifestyle therapy	%TBWL (12 mth): significantly greater in the ESG vs. SMT groups (20.6 vs. 14.3, *p* < 0.001) However, no significant difference in weight loss was observed between ESG or SMT at 12 months for morbidly obese patients with initial BMI >40

*%TBWL, percentage total body weight loss; BMI, body mass index; T2DM, type 2 diabetes mellitus; FPG, fasting plasma glucose; TG, triglycerides; LDL, low density lipoprotein*.

Notwithstanding the large sample size of both studies with more than 300 participants, further longitudinal studies will be required to characterize the effects of ESG on weight in comparison to realistic first-line SMT. It should be noted that the SMT in the Sullivan study was low-intensity lifestyle therapy and sham procedure, including only 6 lifestyle therapy visits during the 12-month follow-up.

#### Effects on Glycemic Control and Cardiometabolic Risk Factors

There is a significant improvement of diabetes, defined as a decrease in diabetes medication, in the ESG group as compared to the control group at 12 months. Trends of reduced hypertension, HDL, LDL and triglycerides were observed to be greater in the ESG group vs. control group, but were not statistically significant, requiring further longitudinal studies for validation.

#### Safety

Procedure-related serious adverse event rates were 5.0% (active) and 0.9% (sham), including extra-gastric bleed, pain, nausea and vomiting. Other general adverse effects are sore throat, heartburn/reflux, mouth trauma and gastric erosion.

#### Comparison With Laparoscopic Surgery

ESG was initially designed as a minimally invasive endoscopic alternative to surgical sleeve gastrectomy, hoping to achieve similar clinical efficacy (27). Case-control studies performed by Abu Dayyeh et al. (35) and Fayad et al. (36) comparing ESG with laparoscopic sleeve gastrectomy (LSG) have concluded that both ESG and LSG achieve significant weight loss, with the caveat that weight loss was lower with ESG [%TBWL: 17.1 vs. 23.6% in the Fayad study (35) and 18.5 vs. 28.3% in the Lopez-Nava study (13)]. Nonetheless, ESG demonstrates lower complication rates for conditions such as new-onset Gastroesophageal Reflux Disease (GERD) and requires a shorter hospital stay compared to LSG.

### Duodenal-Jejunal Bypass Liner

#### Effect on Body Weight

Four randomized controlled trials and one case control study investigated the effects of DJBL on weight loss (Table 5). Large-sample randomized controlled trials (of 170 participants each) conducted by Ruban et al. (37) and Glaysher et al. (38) both showed significantly greater TBWL in the DJBL group vs. SMT group, Three studies comprising 510 participants (21, 38, 40) studied change in BMI between the DJBL and SMT group at 6 months (Figure 2D). Our analysis showed no significant reduction in BMI between the DJBL and SMT group (WMD −2.73 (−5.52, 0.07) kg/m^2^)). Nevertheless, the long-term impact of DJBL on weight loss post-explantation remains uncertain. While Ruban and his colleagues (37) concluded a significant reduction in weight loss at 12 months, there was no significant difference in %TBWL observed between both groups at 24 months after the removal of DJBL. The other study by Caiazzo et al. (39) also found no significant difference in observed BMI loss after the removal of DJBL.

**Table 5:** Studies included for the study of DJBL on Metabolic Syndrome and related comorbidities in adults.

**References**	**Type of study/No. of patients recruited**	**Study group(s)**	**Selection criteria/ Demographics of patients recruited**	**Results/outcome(s) of interest**
Ruban et al. (37)	Open-label randomized controlled trial *n* = 170 (DJBL 85, SMT 85)	DJBL vs. SMT	Patients with BMI of 30–50 kg/m^2^, inadequately controlled T2DM of duration ≥1 year, and oral glucose-lowering medication prescription	>15% TBWL (12 mth): significantly greater in the DJBL vs. SMT groups (24.2 vs. 3.7; *p* = 0.007). No significant difference at 24 months after the removal of DJBL Reduction in HbA1c (24 mth): no significant difference Reduction in blood pressure, total cholesterol, ALT and AST: DJBL significantly greater at 12 months, but no significant difference at 24 months
Glaysher et al. (38)	Randomized controlled trial *n* = 170 (DJBL 85, SMT 85)	DJBL vs. SMT	Patients with T2DM for at least 1 year, BMI 30–50 kg/m^2^, and oral antihyperglycemic medication prescription	%TBWL (11.5 mth): DJBL >SMT (11.3 vs. 6.0; *p* < 0.001) Total cholesterol and LDL cholesterol (6 and 11.5 mth): significantly lower in DJBL
Caiazzo et al. (39)	Randomized controlled trial *n* = 80 (DJBL 49, SMT 31)	DJBL vs. SMT	Patients with BMI >30 kg/m^2^, and a clinical diagnosis of MS	BMI loss: more significant in the DJBL group while DJBL was in place, no significant difference after removal HbA1c: DJBL >SMT, no difference after removal HOMA2, blood pressure, HDL cholesterol and TG: no difference after removal
Laubner et al. (40)	Retrospective case-matched study *n* = 333 (DJBL 111, Control 222)	DJBL vs. SMT	Patients with T2DM and BMI >27 kg/m^2^	BMI loss: DJBL >SMT (5.31 vs. 0.39; p < 0.0001) HbA1c, blood pressure, total cholesterol, LDL cholesterol: significantly greater in the DJBL vs. SMT groups
Koehestanie et al. (41)	Randomized controlled trial *n* = 77 (DJBL 38, Control 39)	DJBL vs. SMT	Patients with T2DM and BMI ≥30 kg/m^2^	%EBWL (12 mth): significantly greater in the DJBL than control group (19.8 vs. 11.7; *p* < 0.05) HbA1c (12 mth): no significant difference

*%TBWL, percentage total body weight loss; BMI, body mass index; T2DM, type 2 diabetes mellitus; HbA1C, glycated hemoglobin; TG, triglycerides; LDL, low density lipoprotein; HDL, high density lipoprotein; EFA, essential fatty acid; HOMA2, homeostatic model assessment for insulin resistance; ALT, alanine aminotransferase; AST, aspartate aminotransferase*.

#### Effects on Glycemic Control and Cardiometabolic Risk Factors

There were four studies that examined the effect of DJBL on HbA1c compared with SMT (Table 5). The effect of treatment with DJBL on glycemic control remains ambiguous. The Caiazzo study (39) and Laubner study (40) both found that HbA1c was significantly decreased in the DJBL group as compared to the SMT group, while the Koehestanie et al. (41) and Ruban et al. (37) studies observed no significant difference between the 2 groups in achieving a reduction in HbA1c at 12 and 24 months respectively. Out of the four studies, three (37, 39, 40) of them with a total of 507 participants assessed the effect of DJBL on %HbA1c change (Figure 2E). Overall, the DJBL group in these studies experienced a greater reduction in %HbA1c at 12 months as compared to the SMT group, with a WMD of −0.54% (−0.88, −0.20).

Similarly, for the other cardiometabolic risk factors such as blood pressure, EFAs, total cholesterol, and LDL cholesterol, Laubner et al. (40) and Glaysher et al. (38) both showed significant decreases in the DJBL group vs. the SMT group. However, Caiazzo et al. (39) found that there was no significant difference in BMI loss, HOMA2, blood pressure, HDL cholesterol and triglycerides after removal of DJBL. Additionally, with respect to cardiovascular risk profile, Koehestanie et al. (41) found that DJBL intervention could possibly reduce the estimated 10-year coronary heart disease risk by 2 vs. 1% in the control group according to the UK Prospective Diabetes Study Risk Engine (42).

#### Safety

Caiazzo et al. (39) reported 39% of patients who received DJBL experienced at least 1 SAE related to the device. Twenty-two percent required surgical or endoscopic intervention and 16% of the subjects required premature removal of the device, due to occlusion, device migration, abdominal pain, and gastrointestinal hemorrhage. Other adverse events include abdominal pain, gastrointestinal bleeding, device occlusion, and musculoskeletal injury. Ruban et al. (37) reported one case of liver abscess related to DJBL.

### Duodenal Mucosal Resurfacing

#### Effect on Body Weight

Two studies involving DMR, both randomized controlled trials, were included in this systematic review (Table 6). Given the small total number of participants included in both studies, it is difficult to conclude if DMR produces a significant weight-lowering effect. The study by Mingrone et al. (43) was conducted across 11 sites in Europe and Brazil and included 108 participants. The study saw a significantly greater reduction in weight of 2.4 kg in participants who received DMR as compared to SMT in the European population at 24 weeks. However, this benefit of weight reduction was not seen in the Brazilian population. This was explained by the suggestion that the Brazilian population received more intensive SMT than the European population. Thus, although the weight loss in the Brazilian population was greater at 4.1 kg, it was not significantly greater than that due to SMT. Another study by Kaur et al. (44) involving 30 participants found no significant weight reduction benefit from DMR over SMT.

**Table 6:** Studies included for the study of DMR on Metabolic Syndrome and related comorbidities in adults.

**References**	**Type of study/No. of patients recruited**	**Study group(s)**	**Selection criteria/ Demographics of patients recruited**	**Results/outcome(s) of interest**
Mingrone et al. (43)	Double-blind multicenter randomized sham-controlled trial; *n* = 108 (DMR 56, SMT 52)	DMR vs. SMT	Patients with HbA1c 59–86 mmol/mol, BMI ≥24 and ≤40 kg/m^2^, fasting insulin >48.6 pmol/L and on ≥1 oral antidiabetic medication	EWL (24 weeks): significantly greater in the DMR vs. SMT group (2.4kg vs. 1.4 kg; *p* = 0.012) in the European population. No significant difference in the Brazilian population HbA1c: more significantly reduced with DMR vs. SMT (6.6 mmol/mol vs. 3.3 mmol/mol; *p* = 0.033) in the European population. In the Brazilian population, this effect was only seen with a mixed-model repeated measures analysis Liver fat (MRI-PDFF): Greater reduction of liver fat content in the DMR group (5.4% vs. 2.4%; *p* = 0.035). This effect was not seen in the Brazilian population
Kaur et al. (44)	Multicentre randomized double-blind sham-controlled trial; n=32 (DMR 16, SMT 16)	DMR vs. SMT	Women of reproductive potential aged between 18 and 50 years, BMI ≥30 kg/m^2^, diagnosis of polycystic ovary syndrome, insulin resistance and <6 reported menses in the 12 months prior to screening	Weight loss (24 weeks): minimal in DMR and SMT groups with no significant difference between groups Insulin sensitivity (HOMA-IR): Increased non-significantly in both groups Glucose and insulin excursion: no significant difference between groups at 3 months Lipids: no significant difference between groups Liver profile: no significant difference between groups

*EWL, excess weight loss; BMI, body mass index; HbA1c, glycated hemoglobin; HOMA-IR, homeostatic model assessment for insulin resistance; MRI-PDFF, magnetic resonance imaging proton density fat fraction*.

#### Effects on Glycemic Control and Cardiometabolic Risk Factors

The European DMR group in the Mingrone et al. study (43) saw a significantly greater reduction in HbA1c of −6.6 mmol/mol as compared to −3.3 mmol/mol in the sham control group. In the Brazilian population, the reduction in HbA1c in participants who received DMR was not significantly greater than those that received SMT although the reduction was as high as −20.2 mmol/mol at week 24. The decrease in FPG in both populations was not significantly different between the DMR and the SMT groups.

In the Kaur et al. study (44) which measured changes in insulin resistance, there was no significant difference between DMR and SMT groups in terms of insulin sensitivity as measured by HOMA-IR and insulin and glucose excursions.

#### Effects on NAFLD

Liver MRI-PDFF changes were used to measure liver steatosis levels in the Mingrone et al. study. The median change in liver MRI- PDFF from baseline at 12 weeks demonstrated a reduction of liver fat content by 6.1% in the DMR group compared with 4.3% in the sham control group (*p* = 0.035; treatment difference −3.2%) (35). However, Kaur et al. (44) found no significant difference in the liver profile of participants from the DMR and the control group.

#### Safety

Most adverse events were mild and transient, including abdominal pain, diarrhea, nausea, vomiting, and hypoglycemia.

### Risk of Bias and Quality of Evidence

The Cochrane RoB 2 and ROBINS-I tools were applied to evaluate the risk of bias for RCTs and non-randomized studies respectively. We identified a low risk of bias in all the RCTs selected (Table 7), with the exception of several RCTs exhibiting some concerns of bias arising from the randomization process. This was most commonly due to the studies not specifying the method of sequence generation used. Bias arising from missing outcome data was also identified as a significant proportion of studies lost more than 5% of their participants to follow-up. For the included non-randomized studies, these were mostly assessed to have a moderate risk of bias, with one retrospective study deemed to have a serious risk of bias (Table 8). This was as all four studies were judged to have a moderate risk of confounding bias. In addition, the retrospective study was also found to have a serious risk of bias for the domain of selection of participants as it may have excluded data from participants who had an early explantation of the DJBL.

**Table 7:** Risk of bias assessment for included randomized controlled trials.

**First author**	**1.1**	**1.2**	**1.3**	**2.1**	**2.2**	**2.3**	**2.4**	**2.5**	**2.6**	**2.7**	**3.1**	**3.2**	**3.3**	**3.4**	**4.1**	**4.2**	**4.3**	**4.4**	**4.5**	**5.1**	**5.2**	**5.3**	**R**	**D**	**Mi**	**Me**	**S**	**O**
Courcoulas, A.	Y	Y	N	Y	Y	N	NA	NA	Y	NA	Y	NA	NA	NA	N	N	Y	N	NA	Y	N	N	L	L	L	L	L	L
Sullivan, S.	Y	Y	N	Y	N	N	NA	NA	Y	NA	N	N	N	NA	N	N	Y	N	NA	Y	N	N	L	L	L	L	L	L
Ponce, J.	Y	Y	N	Y	N	N	NA	NA	Y	NA	N	N	PN	NA	N	N	N	NA	NA	Y	N	N	L	L	L	L	L	L
Chan, DL.	NI	Y	N	Y	Y	N	NA	NA	Y	NA	N	N	NI	N	N	N	Y	N	NA	NI	N	N	L	L	SC	L	L	SC
Coffin, B.	Y	Y	N	Y	Y	N	NA	NA	Y	NA	N	N	PN	NA	N	N	Y	N	NA	Y	N	N	L	L	L	L	L	L
Farina, MG.	NI	NI	N	Y	Y	N	NA	NA	Y	NA	N	N	N	NA	N	N	Y	N	NA	NI	N	N	SC	L	L	L	L	SC
Lee, YM.	Y	Y	N	Y	Y	N	NA	NA	Y	NA	N	N	N	NA	N	N	Y	N	NA	Y	N	N	L	L	L	L	L	L
Mohammed, AM.	NI	NI	N	Y	Y	N	NA	NA	Y	NA	Y	NA	NA	NA	N	N	Y	N	NA	Y	N	N	SC	L	L	L	L	SC
Fuller, NR.	PY	PY	N	Y	Y	N	NA	NA	Y	NA	N	Y	NA	NA	N	N	Y	N	NA	NI	N	N	L	L	L	L	L	L
Martin, CV.	Y	Y	N	Y	N	N	NA	NA	Y	NA	N	Y	NA	NA	N	N	Y	N	NA	Y	N	N	L	L	L	L	L	L
Ruban, A.	Y	Y	N	Y	Y	N	NA	NA	Y	NA	N	Y	NA	NA	N	N	Y	N	NA	Y	N	N	L	L	L	L	L	L
Glaysher, MA.	Y	Y	N	Y	Y	N	NA	NA	Y	NA	N	Y	NA	NA	N	N	PY	N	NA	Y	N	N	L	L	L	L	L	L
Caiazzo, R.	Y	Y	N	Y	Y	N	NA	NA	Y	NA	N	Y	NA	NA	N	N	Y	N	NA	PY	N	N	L	L	L	L	L	L
Koehestanie, P.	PY	PY	N	Y	Y	N	NA	NA	Y	NA	N	N	PN	NA	N	N	Y	N	NA	Y	N	N	L	L	L	L	L	L
Mingrone, G.	Y	Y	N	N	N	N	NA	NA	Y	NA	Y	NA	NA	NA	N	N	Y	N	NA	Y	N	N	L	L	L	L	L	L
Kaur, V.	Y	Y	N	N	N	N	NA	NA	Y	NA	Y	NA	NA	NA	N	N	Y	N	NA	Y	N	N	L	L	L	L	L	L
Sullivan, S.	Y	Y	N	N	N	N	NA	NA	Y	NA	Y	NA	NA	NA	N	N	Y	N	NA	Y	N	N	L	L	L	L	L	L

*Y, Yes; N, No; PY, Probably yes; PN, Probably no; NI, No information; NA, Not applicable; L, Low; SC, some concerns; R, Bias arising from the randomization process; D, Bias due to deviations from intended interventions; Mi, Bias due to missing outcome data; Me, Bias in measurement of the outcome; S, Bias in selection of the reported result; O, Overall risk of bias. columns were colour-coded according to the relevant domain of bias*.

**Table 8:** Risk of bias assessment for included non-randomized studies.

**First author**	**Co**	**P**	**Ca**	**D**	**Mi**	**Me**	**R**	**O**
Mariani, S.	Moderate	Low	Low	Low	Low	Low	Low	Moderate
Takihita, M.	Moderate	Low	Low	Low	Low	Low	Low	Moderate
Laubner, K.	Moderate	Serious	Moderate	Low	Low	Low	Low	Serious
Cheskin, LJ.	Moderate	Moderate	Moderate	Low	Low	Low	Low	Moderate

*Co, Bias due to confounding; P, Bias in selection of participants into the study; Ca, Bias in classification of interventions; D, Bias due to deviations from intended interventions; Mi, Bias due to missing data; Me, Bias in measurement of outcomes; R, Bias in selection of the reported result; O, Overall risk of bias. columns were colour-coded according to the relevant domain of bias*.

## Discussion

We conducted a systematic review and meta-analysis of the four promising endoscopic bariatric metabolic therapies – intragastric balloon, endoscopic sleeve gastroplasty, duodenal jejunal bypass liner, and duodenal mucosal resurfacing. They fill an important gap between pharmacotherapy and bariatric surgery by offering subjects who have failed lifestyle and medical therapies but are not willing or fit enough to go for major surgery. Our review covers the most current available literature and focuses on high-quality studies that include an appropriate control study group, to evaluate the benefits and risks of each modality in NAFLD and in the control of the accompanying cardiovascular risk factors.

To date, IGB is one of the most widely studied EBMTs, as it was one of the first EBMTs conceptualized. IGB showed promising results on total body weight loss at 6 months, although there was an increase in participant's weight 6 months post-removal of IGB, suggesting limitations in the durability of weight loss. The concept of weight recidivism after IGB removal was also reviewed by Tate and Geliebter (45), suggesting that most patients would have difficulty maintaining weight loss following the standard 6 months treatment with IGB. However, there is still a significant effect on total body weight with IGB compared to the standard medical therapy group. The American Society for Gastrointestinal Endoscopy (ASGE) Bariatric Endoscopy Task Force (46) concluded that IGB therapy resulted in 25% of excess weight loss at 12 months, recommending IGB as a suitable EBMT of choice either as a primary EBMT modality, or bridging therapy to eventual bariatric surgery. However, most IGBs are designed to last for 6 months, and there is a paucity of data on the effect of repeated placement of IGB.

From our systematic review of the four modalities, IGB, ESG and DJBL show promising benefits on weight loss reduction compared to SMT, while DMR appears to have the least weight reduction benefit. However, the impact on glycemic control features more prominently in DMR as compared to the rest of the modalities. Van Baar et al. (47) conducted a multi-centered, open-label study of 46 patients and found significant improvements in HbA1c, FPG, and HOMA-IR 6 months post DMR with sustained effects at 12 months. Hepatic transaminase levels also decreased. However, it was noted that change in HbA1c did not correlate with weight loss. As DMR is a relatively new EBMT developed, more prospective controlled clinical trials are required for validation given the current paucity of high-quality studies.

Our meta-analysis focused on two main outcomes in the IGB and DJBL intervention group: weight loss (%TBWL and BMI) and glycemic control (FPG, HbA1c). Overall, our results suggest that participants treated with IGB showed superior results in both weight loss and glycemic control compared to SMT alone, while DJBL showed a positive impact on glycemic control but not on weight loss reduction. There were a large number of patients included in each analyses, with more than 400 patients analyzed for each outcome. This is despite excluding some studies that lacked available data for the calculation of standard deviation. In general, the large number of patients analyzed makes this a robust analysis.

There is inconclusive evidence on the overall impact of the four EBMTs on cardiometabolic risk factors (systolic blood pressure, total cholesterol, triglycerides). This is likely because BMI was featured as the main patient selection criteria (primary endpoint), with hypertension, diabetes, and dyslipidemia featured as the secondary endpoints. For example, the baseline prevalence of type II DM was 7% and 6% in the IGB and SMT groups respectively in the study conducted by Courcoulas et al. (21). Future studies focusing on cardiometabolic risk as a primary endpoint should be conducted to draw more conclusive evidence.

Additionally, there was a general lack of high-quality studies looking at the impact of each EBMT on liver biochemistries or NASH as many did not include an appropriate control arm. From our literature review of existing studies that did not include a control arm, both IGB and ESG show promising effects on NASH and liver biochemistries. We identified one unique pilot randomized, controlled trial of IGB vs. SMT in NAFLD subjects, with repeat liver biopsy, which showed a trend toward improvement in the median steatosis scores after 6 months. However, the sample size is too small to be conclusive. A prospective study conducted by Bazerbachi et al. (48) analyzed the impact of IGB on metabolic and histologic improvements in nonalcoholic steatohepatitis (NASH) and found that the nonalcoholic fatty liver disease activity score (NAS) improved in 90% of the patients, with a median decrease of three points. Similarly, prospective studies conducted by Jagtap et al. (49), Hajifathalian et al. (50), Rosenblatt et al. (51) showed that ESG had significant improvement in ALT, NAFLD fibrosis and FIB-4 score and hepatic steatosis index (HSI). There are only two studies in our systematic review that looked at the impact of DMR on liver biochemistry by Mingrone et al. (43) and Kaur et al. (44), and both concluded that DMR has no significant difference in the liver profile.

There is a paucity of data comparing EBMTs and their effects on weight loss and metabolic syndrome parameters. Available literature mainly featured IGB vs. ESG. Fayad et al. (52) conducted a retrospective review of prospectively collected data analyzing the outcomes of IGB and ESG, and found that IGB patients showed a significantly lower mean %TBWL than ESG patients at 1 month (6.6 vs. 9.9 %), 3 months (11.1 vs. 14.3 %), 6 months (15.0 vs. 19.5 %), and 12 months (13.9 vs. 21.3 %). The study also concluded that IGB had a significantly greater rate of adverse events compared with the ESG group (17 vs. 5.2%). However, no adverse events required surgical intervention in both groups and there was no reported mortality with either procedure.

Device-related adverse events featured more prominently in DJBL. In 2015, the US ENDO trial was terminated due to a higher than expected hepatic abscess rate (3.5%) compared with the global incidence (0.73%) (53). Caiazzo et al. (39) observed device-related severe adverse events in about 39% of the patients, which led to premature removal of the device in 22% of them. Given the lower safety profile compared to the rest of the EBMTs, the present versions of DJBL are less favored in the clinical setting.

Intrinsic limitations of this systematic review and meta-analysis are evaluated. Firstly, although our comprehensive search of the electronic databases allowed us to identify a large number of articles (a total of 648 records screened), our review eventually only included a total of twenty-one studies. Many studies are excluded due to the lack of a proper control arm, which should include lifestyle modifications for weight loss and physical exercises. Secondly, there may be an element of publication bias for positive outcomes over those with negative results. Thirdly, some studies could not be included because of language barriers, for example, non-English literature was excluded and some articles could not be accessed fully due to access limitations. Lastly, although a large number of patients were included in our meta-analysis, there was modest to substantial heterogeneity observed. This is likely because of the different BMI selection criteria of patients in each study and most studies only analyzing diabetes as a secondary outcome. As mentioned earlier, the baseline prevalence of type II diabetes mellitus was <10 percent in the study by Courcoulas et al. (21).

Finally, none of these EBMTs exist alone in silos. EBMTs could be recommended as a short-term booster weight-loss strategy, but standard medical therapy such as adequate physical exercise and dietary modifications must also be strictly reinforced to achieve and sustain the desired outcomes of weight loss, reduction in glycemic control and improvement in cardiometabolic parameters. Given human variability, compliance to the recommended dietary or exercise regimens will always be in question, and patient factors eventually play a large role in battling obesity and metabolic syndrome. However, when EBMTs are utilized in complementarity with physician supervision and lifestyle therapy, they offer exciting new opportunities and the much-needed obesity armamentarium for many patients who prefer minimally invasive options.

## Conclusion

In this systematic review and meta-analysis, we summarized the evidence behind the four EBMT modalities (IGB, ESG, DJBL, and DMR) and concluded that they have a significant impact on weight loss and emerging evidence on obesity-related metabolic comorbidities. Both IGB and ESG are more widely used due to their relatively greater safety profile and significant impact on weight loss. The limitations of IGB lie in the durability of its weight loss effect post removal and eventual long-term benefit on cardiovascular outcomes. Both modalities also show emerging evidence in their benefits on NASH, although more studies with control groups can be conducted for higher-quality evidence. DJBL has more commonly reported severe adverse events, lacks sufficient controlled studies that demonstrate robust results, and is thus less commonly recommended in clinical practice. DMR is a new development and seems to demonstrate potential in the improvement of glycemic control, although more data is required before it can be recommended as a standard treatment modality. Newer and better EBMT modalities can be expected to emerge in the coming years to fill the unmet need for safe and effective minimally invasive therapies for NAFLD and metabolic syndrome.

## Data Availability Statement

The original contributions presented in the study are included in the article/supplementary material, further inquiries can be directed to the corresponding author.

## Author Contributions

S-YL is responsible for data analysis and preparation of manuscript. HL and YC performed the literature search, data collection, and data analysis. MW carried out the data analysis for the meta-analysis, completed part of the methodology, and result sections of the manuscript. G-HL is the corresponding author responsible for the study design, data analysis, final manuscript preparation, and submission. All authors contributed to the article and approved the submitted version.

## Conflict of Interest

The authors declare that the research was conducted in the absence of any commercial or financial relationships that could be construed as a potential conflict of interest.

## Publisher's Note

All claims expressed in this article are solely those of the authors and do not necessarily represent those of their affiliated organizations, or those of the publisher, the editors and the reviewers. Any product that may be evaluated in this article, or claim that may be made by its manufacturer, is not guaranteed or endorsed by the publisher.

## Appendix

**Table A1:** List of search terms.

**Modality**	**Search term(s)**
All	1. Endoscopic bariatric and metabolic therapies AND fatty liver 2. Endoscopic bariatric and metabolic therapies AND cardiovascular disease 3. Endoscopic bariatric and metabolic therapies AND metabolic syndrome 4. Endoscopic bariatric and metabolic therapies AND atherosclerosis 5. Endoscopic bariatric and metabolic therapies AND hyperlipidemia 6. Endoscopic bariatric and metabolic therapies AND hypertension 7. Endoscopic bariatric and metabolic therapies AND diabetes mellitus
IGB	8. Gastric balloon AND fatty liver 9. Gastric balloon AND cardiovascular disease 10. Gastric balloon AND metabolic syndrome 11. Gastric balloon AND atherosclerosis 12. Gastric balloon AND hyperlipidemia 13. Gastric balloon AND hypertension 14. Gastric balloon AND diabetes mellitus
ESG	15. Endoscopic sleeve gastroplasty AND fatty liver 16. Endoscopic sleeve gastroplasty AND cardiovascular disease 17. Endoscopic sleeve gastroplasty AND metabolic syndrome 18. Endoscopic sleeve gastroplasty AND atherosclerosis 19. Endoscopic sleeve gastroplasty AND hyperlipidemia 20. Endoscopic sleeve gastroplasty AND hypertension 21. Endoscopic sleeve gastroplasty AND diabetes mellitus
DJBL	22. Duodenal-Jejunal Bypass Liner AND fatty liver 23. Duodenal-Jejunal Bypass Liner AND cardiovascular disease 24. Duodenal-Jejunal Bypass Liner AND metabolic syndrome 25. Duodenal-Jejunal Bypass Liner AND atherosclerosis 26. Duodenal-Jejunal Bypass Liner AND hyperlipidemia 27. Duodenal-Jejunal Bypass Liner AND hypertension 28. Duodenal-Jejunal Bypass Liner AND diabetes mellitus 29. Endobarrier AND fatty liver 30. Endobarrier AND cardiovascular disease 31. Endobarrier AND metabolic syndrome 32. Endobarrier AND atherosclerosis 33. Endobarrier AND hyperlipidemia 34. Endobarrier AND hypertension 35. Endobarrier AND diabetes mellitus
DMR	36. Duodenal mucosal resurfacing AND fatty liver 37. Duodenal mucosal resurfacing AND cardiovascular disease 38. Duodenal mucosal resurfacing AND metabolic syndrome 39. Duodenal mucosal resurfacing AND atherosclerosis 40. Duodenal mucosal resurfacing AND hyperlipidemia 41. Duodenal mucosal resurfacing AND hypertension 42. Duodenal mucosal resurfacing AND diabetes mellitus
